# Recent Advances in Fish Nutrition: Insights on the Nutritional Implications of Modern Formulations

**DOI:** 10.3390/ani12131705

**Published:** 2022-07-01

**Authors:** Artur Rombenso, Bruno Araujo, Erchao Li

**Affiliations:** 1 Livestock and Aquaculture Program, Agriculture & Food, CSIRO, Woorim, QLD 4507, Australia; 2 Cawthron Institute, Aquaculture Program, Nelson 7010, New Zealand; bruno.araujo@cawthron.org.nz; 3 Department of Aquaculture, College of Marine Sciences, Hainan University, Haikou 570228, China; ecli@bio.ecnu.edu.cn

Fish nutrition has driven advances in the efficiency, sustainability, and product quality of aquaculture production, facilitating its expansion of aquaculture production [1,2]. Formulations have evolved due to the immense R&D effort of academic and commercial sectors, as well as regulatory pressures. For example, modern diets can maximise production performance, facilitate immune resilience, and achieve a tailored edible product, while also meeting acceptable social licenses and minimising the environmental footprint. More recently, the pandemic has expediated the adoption of new feeds and raw materials for use in fish production as a way of mitigating the rising volatile prices and availability of traditional ingredients. The aquafeed industry has sought more flexible formulations by continuing searching, testing, and adopting novel products and considering locally sourced ingredients while maintaining feed composition and performance, even though some might not be as price competitive as overseas ones. Some major trends for commercial application and R&D include high-energy diets, satisfying nutritional needs beyond the species requirements, benefiting animal physiology and welfare, addressing sustainability standards, novel ingredients and feed additives, and improving the nutritional quality of the final product.

The present Special Issue “Recent Advances in Fish Nutrition: Insights on the Nutritional Implications of Modern Formulations” ambitiously aimed to present the latest advances in fish nutrition based on modern formulations that remarkedly differ from traditional ones. The topics covered can be divided into six focus areas: (1) the reassessment of macronutrients (protein, lipid, and carbohydrate) demands; (2) the reassessment of essential nutrients (amino acids, fatty acids, vitamins, minerals, and others); (3) the use of feed additives; (4) novel ingredients, including alternative ingredients, complementary ingredients, and blends of ingredients; (5) organic feeds; and (6) nutritional quality of the final product.

This Special Issue features eleven articles from different parts of the globe, including research centres and universities in Australia, Brazil, Canada, China, Cotonou, Egypt, Japan, Saudi Arabia, Spain, and New Zealand, with a variety of fish species, such as red seabream Pagrus major, rainbow trout Oncorhynchus mykiss, Nile tilapia *Oreochromis niloticus*, common carp *Cyprinus carpio*, rice field eel *Monopterus albus*, Japanese sea bass *Lateolabrax japonicus*, Asian seabass *Lates calcarifer*, and dusky grouper *Epinephelus marginatus*. Most articles focus on feed additives and their effects on production performance, immunity, and stress [3,4,5,6]. Novel ingredients, including camelina meal and *Schizochytrium* sp., were investigated as complementary ingredients in the context of production performance, immune and stress response, digestibility, digestive enzyme activity, and transcript expression [7,8,9]. Nutrient sources with distinct chemical forms, digestibility, and metabolism, and the resulting environmental footprints, are also discussed [10,11]. Other relevant research topics include insights into the underlying mechanisms of appetite and the interaction between culture conditions and fatty acid metabolism [4,12].

Marine-origin ingredients (i.e., fishmeal (FM) and fish oil (FO)) continue to be strategic components of aquafeeds, but at lower inclusion levels than decades ago. However, high and volatile prices, limited availability, composition variability, social license, and nitrogen pollution can be considered potential challenges faced by FM- and FO-rich feeds [13,14,15,16,17]. A global research effort has been made in the last three decades seeking complementary ingredients in aquafeeds [1]. One of the most viable and promising sources is camelina meal. In a study with red seabream (*Pagrus major*) juveniles, the replacement of FM by 205 g/kg camelina meal resulted in a consistent performance, higher digestive enzyme activities, and improved nutrient digestibility, stress resistance, and immune systems compared with fish fed an FM-based diet [7].

The main challenge in replacing marine-origin ingredients relates to the essential omega 3 long-chain polyunsaturated fatty acid (n-3 LC-PUFA) levels, since alternative lipid sources, mainly vegetable oils and terrestrial animal fats, which are commonly used by the aquafeed industry, do not present significant levels of these important nutrients [1,2]. The most physiologically relevant n-3 LC-PUFA for fish is docosahexaenoic acid (DHA, 22:6n-3), which plays several essential functions, such as the development of visual and nervous tissue and immunological system modulation, in addition to being highly relevant to human nutrition. Thus, it is paramount to consider and address dietary DHA content when using complementary sources to successfully replace FO. Recent studies have shown that microalgae products (e.g., meal and oil) can be considered one of the most promising types of ingredients to replace FO. In this Special Issue, two studies focusing on FO replacement by marine microalgae oil (*Schizochytrium* sp.) in juvenile rainbow trout were published [8,9]. In the first study, the effects of graded algal oil inclusion levels on the apparent digestibility of macronutrients at two temperatures (8 °C and 15 °C) were investigated [9]. The results showed that different temperatures and FO replacement by algal oil did not impair lipid and fatty acid (mainly DHA) digestibility. Similarly, the positive results of including algal oil were found [8]. At lower and higher levels, fish oil replacement by *Schizochytrium* sp. oil resulted in consistent growth performance compared to fish fed the FO-based diet. However, it is noteworthy that diets containing lower algal oil inclusion negatively impacted fillet quality, mainly due to lower DHA levels than fish fed higher algal-oil- and FO-based diets. The chemical form of delivery of n-3 LC-PUFA as phospholipids (PL) or triglycerides (TG) might influence larval and juvenile fish performance [11]. The effects of n-3 LC-PUFA supplementation in the form of PL and TG at lower and higher levels on the tissue fatty acid profile and liver morphophysiology of dusky grouper juveniles were investigated [11]. Although growth indexes were not influenced by dietary treatments, distinct n-3 LC-PUFA accumulation in the different polarities of lipids across tissues was noticed. Beyond diet composition, several other biotic and abiotic factors, such as salinity, water temperature, and life stage, can influence fatty acid synthesis and catabolism processes in commercial fish species, consequently changing their composition. For instance, higher n-3 LC-PUFA levels were found in Japanese sea bass cultured in seawater than in fish kept in freshwater [13]. The authors suggest that n-3 LC-PUFA synthesis was especially modulated by the upregulation of lipid-relevant genes such as elongase 5 (*elovl5*) and fatty acid Δ6 desaturase.

As previously mentioned, FM replacement can be considered an important step to improve sustainability and profitability. Thus, nutritionists are working to judiciously integrate this ingredient with complementary plant byproducts. However, high-carbohydrate diets (normally rich in plant byproducts) can result in lipid metabolism disorders that negatively impact gut and overall health, performance, and fish quality. Previous studies have shown that dietary *myo*-inositol (an important growth-promoting factor of animal cells) can significantly reduce hepatic and mesenteric fat levels in fish species. The benefits of dietary *myo*-inositol were noticed in an experiment carried out with tilapia juveniles fed high carbohydrate levels [6]. After 8 weeks of the feeding trial, fish fed *myo*-inositol showed better growth performance and higher protein retention than those fed the control diet (without *myo*-inositol). According to the authors, *myo*-inositol inclusion effectively decreases lipid accumulation induced by high carbohydrate intake, accelerating the transportation of cholesterol back to the liver and promoting lipid catabolism.

FM-rich diets typically result in high levels of phosphorus and nitrogen discharge in water, contributing to eutrophication in aquatic ecosystems. FM replacement by complementary protein sources is a viable alternative to address these negative impacts. However, a dietary phosphorus supply is necessary to meet fishes’ nutritional requirements. Thus, the effects of diets containing four different inorganic phosphorus sources on the digestibility and excretion of rainbow trout juveniles were evaluated [10]. The results showed that diets containing monoammonium phosphate resulted in similar digestibility compared to diets containing monosodium/monocalcium phosphate. However, significantly lower nitrogen and phosphorus excretion was observed, resulting in a more environmentally friendly diet for this important commercial species.

With the high restrictions of several countries on antibiotic use by the aquaculture sector, several studies have been performed aiming to find alternative feed additives to improve immunity/health and consequently the performance and survival of aquaculture species. Components derived from plants and herbs have emerged as eco-friendly alternatives to antibiotics [3]. Two interesting studies published in this Special Issue have highlighted the potential to use plant-derived components to improve immunity, antioxidant capacity, microbiome, gene expression, and, consequently, the performance of commercial fish species. It was suggested that diets containing 75 or 150 mg/kg of andrographolide (a labdane diterpenoid isolated from the leaves and roots of *Andrographis paniculata*) improved the immunity of rice field eel (*Monopterus albus*) juveniles by enhancing antioxidant capacity, modulating the intestinal physical barrier and microbiome, upregulating anti-inflammatory cytokines, and, inversely, downregulating proinflammatory cytokines in the intestine [3]. Apparently, the anti-inflammatory function of andrographolide was mainly related to the suppression of the Toll-like signalling pathway. Similarly, better performance of barramundi juveniles fed diets containing up to 3% *Glycyrrhiza uralensis* (lowering plant native to several Asian countries) was found [4]. According to the authors, this performance improvement (based on growth, immunity, and survival) was mainly related to the expression of immune-related genes in fish liver and kidney. β-glucan can be considered another efficient prebiotic that is constantly used by the aquaculture industry to enhance fish immunity. It was observed that diets with β-glucan inclusion (0.2% and 0.4%) significantly improved the health of tilapia juveniles cultured in a hyperosmotic environment (brackish water) [5]. In general, fish fed the β-glucan diet showed significantly reduced spleen enlargement, decreased red blood cell count, hematocrit, red-cell distribution width, and downregulation of the expression of immune-related genes. Additionally, diets with 0.4% β-glucan optimised the intestinal microbiota of tilapia in brackish water, consequently improving fish health. The use of pre- and probiotics in aquaculture has been largely investigated [18], and their efficacy varies. 

Apelin is an active polypeptide with many biological functions. In mammals, apelin promotes higher feed intake, enhancing immunity and regulating energy balance [19,20]. However, studies testing Apelin in fish species are scarce. Experiments were conducted to test the effects of Pyr-apelin-13 (an active form of apelin) on common carp in vitro (hypothalamus) and in vivo [12]. The results in vitro and in vivo displayed an upregulation of appetite- and growth-related genes, such as *growth hormone receptor* (GHR) and *insulin-like growth factors 1* and *2* (IGF1 and IGF2, respectively), supporting the hypothesis that this orexigenic peptide might regulate the feeding and growth of this important commercial species. 

In conclusion, this Special Issue briefly illustrates some of the key research areas and advances in fish nutrition. Other Special Issues within *Animals* also relate to topics related to fish nutrition [21,22,23,24,25,26,27]. As the aquafeed industry and R&D develop, we will continue to witness the expansion of the literature, which sometimes can be overwhelming yet impressive. We encourage being critical of the scientific rigor of publications and, more importantly, appreciating the research merits regardless of the source. We, the guest editors, would like to welcome you to our Special Issue.

## References

[1] Turchini G.M., Trushenski J.T., Glencross B.D. (2019). Thoughts for the future of aquaculture nutrition: Realigning perspectives to reflect contemporary issues related to judicious use of marine resources in aquafeeds. N. Am. J. Aquacult..

[2] Hardy R.W., Kaushik S.J., Mai K., Bai S.C., Hardy R.W., Kaushik S.J. (2021). Fish nutrition—History and perspectives. Fish Nutrition.

[3] Shi Y., Zhong L., Liu Y., Zhang J., Lv Z., Li Y., Hu Y. (2020). Effects of Dietary Andrographolide Levels on Growth Performance, Antioxidant Capacity, Intestinal Immune Function and Microbioma of Rice Field Eel (*Monopterus Albus*). Animals.

[4] Yang R., Han M., Fu Z., Wang Y., Zhao W., Yu G., Ma Z. (2020). Immune Responses of Asian Seabass *Lates calcarifer* to Dietary *Glycyrrhiza uralensis*. Animals.

[5] Xu C., Suo Y., Wang X., Qin J.G., Chen L., Li E. (2020). Recovery from hypersaline-stress-Induced immunity damage and intestinal-microbiota changes through dietary β-glucan supplementation in Nile tilapia (*Oreochromis niloticus*). Animals.

[6] Zhu J., Pan J., Wang X., Huang Y., Qin C., Qiao F., Qin J., Chen L. (2020). Alleviation of the adverse effect of dietary carbohydrate by supplementation of myo-Inositol to the diet of Nile tilapia (*Oreochromis niloticus*). Animals.

[7] Mzengereza K., Ishikawa M., Koshio S., Yokoyama S., Yukun Z., Shadrack R.S., Seo S., Kotani T., Dossou S., El Basuini M.F. (2021). Growth performance, growth-related genes, digestibility, digestive enzyme activity, immune and stress responses of de novo camelina meal in diets of Red seabream (*Pagrus major*). Animals.

[8] Osmond A.T.Y., Arts M.T., Hall J.R., Rise M.L., Bazinet R.P., Armenta R.E., Colombo S.M. (2021). *Schizochytrium* sp. (T18) oil as a fish oil replacement in diets for juvenile rainbow trout (*Oncorhynchus mykiss*): Effects on growth performance, tissue fatty acid content, and lipid-related transcript expression. Animals.

[9] Bélanger A., Sarker P.K., Bureau D.P., Chouinard Y., Vandenberg G.W. (2021). Apparent digestibility of macronutrients and fatty acids from microalgae (*Schizochytrium* sp.) fed to rainbow trout (*Oncorhynchus mykiss*): A potential candidate for fish oil substitution. Animals.

[10] Milián-Sorribes M.C., Tomás-Vidal A., Peñaranda D.S., Carpintero L., Mesa J.S., Dupuy J., Donadeu A., Macías-Vidal J., Martínez-Llorens S. (2021). Estimation of phosphorus and nitrogen waste in rainbow trout (*Oncorhynchus mykiss*, Walbaum, 1792) diets including different inorganic phosphorus sources. Animals.

[11] De Mello P.H., Araujo B.C., Marques V.H., Branco G.S., Honji R.M., Moreira R.G., Rombenso A.N., Portella M.C. (2022). Long-Chain Polyunsaturated Fatty Acids n−3 (n−3 LC-PUFA) as Phospholipids or Triglycerides Influence on *Epinephelus marginatus* Juvenile Fatty Acid Profile and Liver Morphophysiology. Animals.

[12] Dong X., Wang J., Ji P., Sun L., Miao S., Lei Y., Du X. (2020). Seawater culture increases omega-3 long-chain polyunsaturated fatty acids (n-3 LC-PUFA) levels in Japanese sea bass (*Lateolabrax japonicus*), probably by upregulating Elovl5. Animals.

[13] Tocher D.R. (2003). Metabolism and functions of lipids and fatty acids in teleost fish. Rev. Fish. Sci..

[14] Tocher D.R. (2015). Omega-3 long-chain polyunsaturated fatty acids and aquaculture in perspective. Aquaculture.

[15] Tacon A.G.J., Metian M. (2008). Global overview on the use of fish meal and fish oil in industrially compounded aquafeeds: Trends and future prospects. Aquaculture.

[16] Turchini G.M., Torstensen B.E., Ng W.-K. (2008). Fish oil replacement in finfish nutrition. Rev. Aquac..

[17] Sprague M., Dick J.R., Tocher D.R. (2016). Impact of sustainable feeds on omega-3 long-chain fatty acid levels in farmed Atlantic salmon, 2006–2015. Sci. Rep..

[18] Akhter N., Wu B., Memon A.M., Mohsin M. (2015). Probiotics and prebiotics associated with aquaculture: A review. Fish Shellfish Immun..

[19] Saral S., Alkanat M., Sumer A., Canpolat S. (2018). Apelin-13 increased food intake with serum ghrelin and leptin levels in male rats. Bratisl. Lek. Listy.

[20] Taheri S., Murphy K., Cohen M., Sujkovic E., Kennedy A., Dhillo W., Dakin C., Sajedi A., Ghatei M., Bloom S. (2002). The effects of centrally administered apelin-13 on food intake, water intaked intake, water intake and pituitary hormone release in rats. Biochem. Biophys. Res. Commun..

[21] Fernandez I., Diaz-Rosales P. (2022). Animals Special Issue “Feed ingredients and Fish Mucosal Health”. https://www.mdpi.com/journal/animals/special_issues/Feed_Ingredients_Fish_Mucosal_Health.

[22] Zanuzzo F.S., Frenette A., Santander J. (2022). Animals Special Issue “Immunity Enhancement Technology in Fish Aquaculture”. https://www.mdpi.com/journal/animals/special_issues/immunity_enhancement_technology_fish_aquaculture.

[23] Aragao C., Engrola S., Costas B. (2022). Animals Special Issue “Advances in Dietary Protein Research: Shaping Innovative Feeds for a Sustainable Ocean”. https://www.mdpi.com/journal/animals/special_issues/Advances_in_Dietary_Protein_Research_Shaping_Innovative_Feeds_for_a_Sustainable_Ocean.

[24] Carral J.M., Saez-Royela M. (2022). Animals Special Issue “Fishmeal and Fish Oil Replacement in Aquaculture”. https://www.mdpi.com/journal/animals/special_issues/fishmeal_fish_oil_replacement_aquaculture.

[25] Samaras A., Dimitroglou A. (2022). Animals Special Issue “Physiological Responses in Fishes”. https://www.mdpi.com/journal/animals/special_issues/fish_physiol.

[26] Lutfi E., Velez E.J. (2022). Animals Special Issue “The Future of Aquaculture Research”. https://www.mdpi.com/journal/animals/special_issues/Future_Aquaculture_Research.

[27] Imperatore R., Paolucci M. (2022). Animals Special Issue “Feed Additives in Health and Immunity of Fish”. https://www.mdpi.com/journal/animals/special_issues/feed_health_fish.

